# INDELseek: detection of complex insertions and deletions from next-generation sequencing data

**DOI:** 10.1186/s12864-016-3449-9

**Published:** 2017-01-05

**Authors:** Chun Hang Au, Anskar Y. H. Leung, Ava Kwong, Tsun Leung Chan, Edmond S. K. Ma

**Affiliations:** 1 Division of Molecular Pathology, Department of Pathology, Hong Kong Sanatorium & Hospital, Happy Valley, Hong Kong SAR; 2 Department of Medicine, The University of Hong Kong, Pok Fu Lam, Hong Kong SAR; 3 Department of Surgery, The University of Hong Kong, Pok Fu Lam, Hong Kong SAR; 4 Department of Surgery and Cancer Genetics Center, Hong Kong Sanatorium & Hospital, Happy Valley, Hong Kong SAR; 5 Hong Kong Hereditary Breast Cancer Family Registry, Shau Kei Wan, Hong Kong SAR

**Keywords:** Complex indel, Variant calling, Bioinformatics, Next-generation sequencing

## Abstract

**Background:**

Complex insertions and deletions (indels) from next-generation sequencing (NGS) data were prone to escape detection by currently available variant callers as shown by large-scale human genomics studies. Somatic and germline complex indels in key disease driver genes could be missed in NGS-based genomics studies.

**Results:**

INDELseek is an open-source complex indel caller designed for NGS data of random fragments and PCR amplicons. The key differentiating factor of INDELseek is that each NGS read alignment was examined as a whole instead of “pileup” of each reference position across multiple alignments. In benchmarking against the reference material NA12878 genome (*n* = 160 derived from high-confidence variant calls), GATK, SAMtools and INDELseek showed complex indel detection sensitivities of 0%, 0% and 100%, respectively. INDELseek also detected all known germline (*BRCA1* and *BRCA2*) and somatic (*CALR* and *JAK2*) complex indels in human clinical samples (*n* = 8). Further experiments validated all 10 detected *KIT* complex indels in a discovery cohort of clinical samples. *In silico* semi-simulation showed sensitivities of 93.7–96.2% based on 8671 unique complex indels in >5000 genes from dbSNP and COSMIC. We also demonstrated the importance of complex indel detection in accurately annotating *BRCA1*, *BRCA2* and *TP53* mutations with gained or rescued protein-truncating effects.

**Conclusions:**

INDELseek is an accurate and versatile tool for complex indel detection in NGS data. It complements other variant callers in NGS-based genomics studies targeting a wide spectrum of genetic variations.

**Electronic supplementary material:**

The online version of this article (doi:10.1186/s12864-016-3449-9) contains supplementary material, which is available to authorized users.

## Background

Complex insertions and deletions (indels) are a known class of genetic variation [1] associated with human diseases [2]. Simultaneous deletion and insertion of DNA fragments of different sizes lead to net change in length. No net change in length is also possible in case of contiguous or non-contiguous multiple-nucleotide variants (MNV). Compared with lower-throughput Sanger sequencing, analysis of next-generation sequencing data relies more on bioinformatics algorithms for automated variant calling. Of concern, recent studies revealed the shortcomings of state-of-the-art variant callers that might fail to detect somatic and germline complex indels [3, 4]. Important mutations in key disease driver genes could be missed in NGS-based genomics studies (e.g. somatic *CALR* complex indels in myeloproliferative neoplasms [5] and germline *BRCA1/BRCA2* complex indels in hereditary breast and/or ovarian cancer [6]).

Pindel-C [3] was introduced to detect the complex indels missed by GATK [7] and VarScan [8] but the implementation was not yet publicly available. Amplicon Indel Hunter [9] and ScanIndel [10] were designed for those that led to >5 bp net change in length or soft-clipping, respectively. MAC [11] targeted MNV only by analyzing single nucleotide variant (SNV) calls of primary callers.

Here we present INDELseek, a software that directly calls somatic and germline complex indels from Sequence Alignment/Map (SAM/BAM) alignments regardless of net change in length.

## Implementation

The INDELseek algorithm was implemented as a single Perl script indelseek.pl that scans each NGS read alignment and identifies closely spaced substitutions, insertions or deletions *in cis* as potential complex indel regardless of net change in length. The only external dependency is SAMtools version 1.3 or above [12], which supports sequencing depth exceeding 8000X in case of deep amplicon sequencing. It was tested on both CentOS Linux 5.5 and Cray XC30 supercomputer (Extreme Scalability Mode) and can be run on the built-in Perl 5 installation of any Linux/Unix-like environment. Alignments of NGS reads in the *de facto* SAM/BAM format [12] serve as input while any complex indel calls will be reported in variant call format (VCF) version 4.1 [13].

**Fig. 1:**
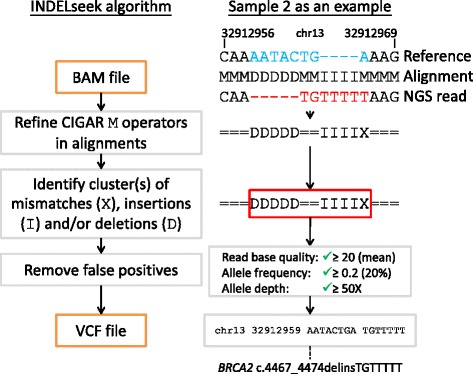
INDELseek algorithm as illustrated by the *BRCA2* complex indel of sample 2. *Left:* INDELseek directly reads NGS read alignments in the standard SAM/BAM format. After refining matches and mismatches in the supplied alignments, clusters of closely spaced mismatches, insertions and/or deletions are identified as potential complex indel calls. False positives are removed according to filters based on read base quality, allele frequency and allele sequencing depth. Final complex indel calls are reported in the standard VCF format. *Right*: A representative BWA-MEM alignment of a sample 2 NGS read was shown. The corresponding reference sequence (chr13:g.32912956_32912969) and base calls of the read were shown above the below the alignment, respectively. In the alignment refinement step, M operators were refined as matches (=) and mismatches (X). A cluster of closely spaced variants was identified as a potential complex indel call (highlighted as a red box). The complex indel call passed the defined quality thresholds and was reported as a variant call in VCF format, which corresponds to the *BRCA2* complex indel c.4467_4474delinsTGTTTTT

INDELseek was designed to identify complex indel(s) at single read level by examining each alignment as a whole (Fig. 1). In contrast, mainstream NGS variant callers examined each reference position across multiple alignments (also known as “pileup”), losing the haplotype information in case of multiple differences compared to reference. Mainstream NGS read aligners (e.g. BWA-MEM) usually align complex indels as multiple mismatches, insertions and/or deletions (Concise Idiosyncratic Gapped Alignment Report (CIGAR) operations M, I and D, respectively) clustered within a short window of reference/read positions, which INDELseek was designed to detect. Since CIGAR operation M could represent either match or mismatch, it was first refined as operations = for match and X for mismatch. INDELseek considers each window fulfilling all of these criteria as a complex indel call: (1) containing at least two X, I and/or D operations that are at most *l* nucleotides away from each other; (2) length at least two nucleotides. The parameter *l* is five by default and is configurable through option --max_distance. For enhanced specificity, false positives can be marked and/or removed based on configurable filters of read base quality, allele frequency and allele depth.

INDELseek parameters were --skip_lowqual --skip_lowdepth --skip_lowaf --min_af 0.2 for germline *BRCA1* and *BRCA2* mutations, --skip_lowqual --skip_lowdepth --skip_lowaf --min_af 0.02 for somatic *CALR, JAK2* and *KIT* mutations, and --skip_lowqual --skip_lowdepth --skip_lowaf --max_distance 10 --min_af 0.2 --min_depth 20 for NA12878 whole-genome sequencing (WGS) dataset. A single CPU core (2010 Intel Xeon X5660 2.8GHz) was measured to be capable to process 56,000 alignments per minute (275 bp MiSeq sequencing reads).

## Results

**Fig. 2:**
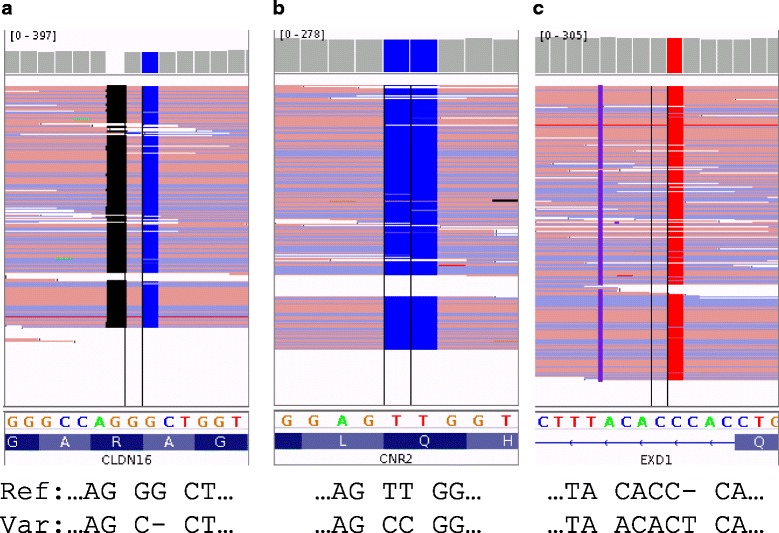
Types of complex indels detected by INDELseek. **a** Net deletion of bases (e.g. chr3:g.190106073_190106074delGGinsC). **b** No net change in length (e.g. chr1:g.24201919_24201920delTTinsCC). **c** Net insertion of bases (e.g. chr15:g.41483633_41483636delCACCinsACACT). Corresponding alignments of reference (Ref) and variant (Var) sequences are shown

We benchmarked complex indel detection performance of GATK, SAMtools and INDELseek using an external WGS dataset of the HapMap NA12878 genome (Illumina HiSeq 2000) and the corresponding high-confidence variant calls from the Genome in a Bottle (GIAB) Consortium [14]. Although the high-confidence variant calls did not comprise complex indels as individual calls, we observed clusters of closely spaced variants calls that appeared *in cis* in the alignments of individual sequencing reads. Accordingly, 160 such loci from GIAB calls were manually curated as putative complex indels (Additional file 1: Table S1) in the intersection (total length 27 Mb) of GIAB high confidence regions and Consensus Coding Sequence Project protein-coding sequences and 10 bp intronic flanking regions [15]. We also observed closely spaced SNV that appeared *in trans* in the alignments and 26 such loci were manually curated as negative controls for complex indel detection (Additional file 1: Table S2). SAMtools and GATK did not call any complex indel from the putative GIAB complex indels (0 of 160) and negative controls (0 of 26), demonstrating 0% sensitivity and 100% specificity. The results were concordant with recent studies that complex indels were mostly missed by bioinformatics pipelines based on common variant callers [3, 4]. INDELseek called all putative GIAB complex indels (160 of 160) and did not call any from negative controls (0 of 26), demonstrating 100% sensitivity and 100% specificity (Table 1). All three types of complex indels resulting in net deletion of bases, no net change in length, or net insertion of bases were detected (Fig. 2a, b, c, respectively). In the context of complex indel detection, the whole-alignment-based approach of INDELseek was demonstrated to be superior to the conventional “pileup” approach of common variant callers.

**Table 1:** Evaluation of INDELseek complex indel detection performance

Dataset	Sample count and description	Sensitivity	Specificity
Real NGS data			
1. Protein-coding and flanking regions from whole-genome sequencing (random fragments)	1 (NA12878)	100%	100%
	160 putative complex indels		
	26 negative control loci		
2. Hereditary breast and/or ovarian cancer panel (amplicons)	239	100%	100%
	3 positive samples (*BRCA1 n* = 1, *BRCA2 n* = 2)		
	236 negative samples		
3. Myeloid neoplasm panel (amplicons)	23	100%	100%
	5 positive samples (*CALR n* = 4, *JAK2 n* = 1)		
	18 negative samples (NA12878 and 17 healthy controls)		
Semi-simulated data by engineering mutations to real NGS data			
1. Whole-genome sequencing (random fragments)	8671 collected from COSMIC and dbSNP	93.7%	N/A
2. Hereditary breast and/or ovarian cancer panel (amplicons)	237 collected from COSMIC and dbSNP	96.2%	N/A
3. Myeloid neoplasm panel (amplicons)	576 collected from COSMIC and dbSNP	94.6%	N/A

N/A Not applicable

Next, we tested INDELseek using two different NGS datasets of PCR amplicons (Table 1). INDELseek was applied to a hereditary breast and/or ovarian cancer (HBOC) panel dataset of 239 probands [6]. The 4-gene panel targeted germline mutations (Illumina MiSeq). Prior Sanger sequencing revealed that three of the probands carried a unique pathogenic complex indel (*BRCA1 n* = 1 and *BRCA2 n* = 2) while remaining 236 probands were negative for complex indel. INDELseek detected all three complex indels (Table 2; Additional file 2: Figure S1-S3), demonstrating 100% sensitivity and 100% specificity. INDELseek was also applied to a myeloid neoplasm (MN) panel dataset of 23 samples [16]. The 54-gene panel targeted somatic mutations (Illumina MiSeq). From five samples known to carry a unique complex indel (*CALR n* = 4 and *JAK2 n* = 1), INDELseek detected all five complex indels (Table 2; Additional file 2: Figure S4-S8).

**Table 2:** Complex indels detected by INDELseek in human clinical samples

Sample	Gene	Mutation	Allele frequency	Sequencing depth (X)	NGS method	Orthogonal validation
Germline pathogenic mutations in hereditary breast and/or ovarian cancers						
1	*BRCA1*	c.4046_4047delinsA p.Thr1349Lysfs*17	37.9%	730	*	†
2	*BRCA2*	c.4467_4474delinsTGTTTTT p.Lys1489Asnfs*15	74.9%	1272	*	†
3	*BRCA2*	c.8400_8402delinsAAAA p.Phe2801Lysfs*11	33.6%	4141	*	†
Somatic pathogenic mutations in myeloid neoplasms						
4	*CALR*	c.1102_1136delinsT p.Lys368Trpfs*51	40.8%	2274	‡	†
5	*CALR*	c.1154delAinsCTTGTC p.Lys385Thrfs*47	31.9%	2998	‡	†
6	*CALR*	c.1129_1154delinsTGTC p.Lys377Cysfs*46	73.6%	2159	‡	†
7	*CALR*	c.1118_1125delinsCTTG p.Asp373Alafs*56	15.3%	3603	‡	§
8	*JAK2*	c.1620_1627delinsGA p.Ile540_Glu543delinsMetLys	57.7%	4629	‡	†
9	*KIT*	c.1248_1257delinsTTGG p.Thr417_Asp419delinsTrp	39.0%	11109	‡	*
10	*KIT*	c.1248_1256delinsTTTCCG p.Thr417_Asp419delinsPheArg	2.9%	13724	‡	*
	*KIT*	c.1249_1258delinsGGATGGAACT p.Thr417_Arg420delinsGlyTrpAsnTrp	3.3%	13651	‡	*
	*KIT*	c.1250_1258delinsAACCTC p.Thr417_Asp419delinsLysPro	11.9%	13525	‡	*
	*KIT*	c.1251_1258delinsCTCCT p.Tyr418_Arg420delinsSerTrp	2.1%	13376	‡	*
11	*KIT*	c.1250_1256delinsT p.Thr417_Asp419delinsIle	5.7%	7326	‡	§
	*KIT*	c.1251_1257delinsAACA p.Tyr418_Asp419delinsThr	2.2%	7416	‡	§
12	*KIT*	c.1251_1256delinsGGG p.Tyr418_Asp419delinsGly	2.7%	14829	‡	*
13	*KIT*	c.1253_1258delinsCCG p.Tyr418_Arg420delinsSerGly	40.7%	68180	‡	*
14	*KIT*	c.1256_1257delinsGTCTA p.Asp419delinsGlyLeu	17.9%	19042	‡	*

*Microfluidic PCR and MiSeq sequencing

†Sanger sequencing

‡Probe extension/ligation and MiSeq sequencing

§PCR fragment analysis

The general applicability of INDELseek in complex indel detection was further assessed using a wider spectrum of complex indels, which showed different combination of deletion and insertion lengths (375 combinations) and different gene context (>5000 genes). We collected 8671 unique complex indels from public databases dbSNP and COSMIC for semi-simulation by *in silico* engineering of complex indels in real NGS datasets. Base quality scores were kept unchanged or similar to flanking bases depending on the net gain in bases (0 or ≥1, respectively). NGS data of NA12878, a *BRCA1*/*BRCA2* complex indel-negative sample, and a healthy adult were selected for engineering from the WGS, HBOC and MN datasets, respectively. INDELseek demonstrated sensitivities of 93.7% (8124 of 8671) for WGS, 96.2% (228 of 237) for HBOC and 94.6% (545 of 576) for MN (Table 1).

As a discovery cohort, INDELseek was applied to an additional MN panel dataset of 10 core-binding factor leukemia samples that were clinically predicted to be enriched for somatic mutations of *KIT* exon 8 [17]. A total of 10 *KIT* in-frame complex indels were detected from six of the samples (1 – 4 complex indels per sample; Table 2) and verified by orthogonal validation experiments (Additional file 2: Figure S9-S14).

To demonstrate the importance of accurate complex indel detection in clinical settings, we focused on 127 MNV in HBOC genes (part of semi-simulation collection) and compared their variant annotation results (Variant Effect Predictor) in two scenarios: (1) original MNV and (2) decomposing MNV into individual single-nucleotide variant for separate annotation, as if the MNV could not be called as a haplotype. Comparison revealed marked difference in 11 (8.7%) MNV, which showed gained (*n* = 5) or rescued (*n* = 6) protein-truncating effects (Table 3). Without accurate calling of complex indels, these MNV would become false negative or false positive pathogenic mutations, respectively. On the other hand, Variant Effect Predictor was tested to natively support complex indels called by INDELseek in VCF format.

**Table 3:** Gained or rescued protein-truncating effect of complex indels

Gene	Genomic position	Multiple-nucleotide variants (MNV)	Predicted protein change	
			MNV called as a haplotype	MNV called as separate single-nucleotide variants
Gained protein-truncating effect				
*BRCA2*	13:32914101-32914102	c.5609_5610delTCinsAG	p.Phe1870*	p.Phe1870Tyr, p.Phe1870Leu
*BRCA1*	17:41245984-41245987	c.1561_1564delGCAGinsTAAA	p.Ala521*	p.Asp522Asn, p.Ala521Glu, p.Ala521Ser
*BRCA1*	17:41244552-41244553	c.2995_2996delCTinsTA	p.Leu999*	p.Leu999Gln, p.=
*TP53*	17:7578486-7578488	c.442_444delGATinsTGA	p.Asp148*	p.Asp148Glu, p.Asp148Gly, p.Asp148Tyr
*TP53*	17:7578286-7578287	c.562_563delCTinsTA	p.Leu188*	p.Leu188Gln, p.=
Rescued protein-truncating effect				
*TP53*	17:7579366-7579368	c.319_321delTACinsCAA	p.Tyr107Gln	p.Tyr107*, p.Tyr107His
*TP53*	17:7578535-7578536	c.394_395delAAinsTG	p.Lys132Trp	p.Lys132Arg, p.Lys132*
*TP53*	17:7578433-7578434	c.496_497delTCinsGG	p.Ser166Gly	p.Ser166*, p.Ser166Ala
*TP53*	17:7578426-7578431	c.499_503delinsTACCT	p. Gln167_His168delinsTyrLeu	p.His168Leu, p.Gln167His, p.Gln167*
*TP53*	17:7578210-7578212	c.637_639delCGAinsTGG	p.Arg213Trp	p.=, p.Arg213*
*TP53*	17:7577508-7577509	c.772_773delGAinsTT	p.Glu258Leu	p.Glu258Val, p.Glu258*

Bold text indicates predicted protein truncation

## Conclusions

This study showed that common variant callers fail to detect complex indels, a finding consistent with recent studies [3, 4]. We also demonstrated that if complex indels were called as individual variant calls (e.g. breaking down a single MNV to multiple SNV), the gained or rescued protein-truncating effects will be mis-interpreted. INDELseek was demonstrated as an accurate and versatile complex indel caller, which is compatible with somatic and germline genomics studies, NGS data of random fragments and PCR amplicons, and all three classes of complex indels (MNV, net insertion and net deletion). Since INDELseek was implemented as a single Perl script that directly reads SAM/BAM alignments and returns complex indel calls in VCF format, it can be readily incorporated into common bioinformatics workflows without any compilation and installation. INDELseek complements other common variant callers in academic and diagnostic NGS-based genomics studies.

## Methods

### Benchmarking based on reference material

High-confidence variants calls and chromosomal regions of NA12878 corresponded to the high-confidence genotype version 2.19 [14]. Closely spaced variant calls were identified by BEDTools version 2.19.1 [18] (parameters: merge –n –d 9). NA12878 200X whole genome sequencing dataset was retrieved from Illumina Platinum Genomes [19]. NA12878 myeloid neoplasm panel dataset (Illumina TruSight myeloid panel) was retrieved from Illumina BaseSpace [20]. GATK HaplotypeCaller version 3.6 [7] and SAMtools version 1.3 [12] with default parameters were used for variant calling. Concordance comparison of variant calls was assisted by vcfeval tool of RTG Tools version 3.6.2 [21].

### Germline complex indel detection in breast and/or ovarian cancers

A total of 239 clinically high-risk breast and/or ovarian cancer patients from Hong Kong Hereditary and High Risk Breast Cancer Programme were selected for this study. Patients were recruited from January 18, 2007 to December 2, 2015 according to previously described criteria [6]. Three patients carrying germline complex indel mutation in either *BRCA1* or *BRCA2* (confirmed by Sanger sequencing) were regarded as positive controls. Another 236 patients either carrying pathogenic mutation other than complex indel or not carrying any pathogenic mutation in *BRCA1* and *BRCA2* (confirmed by full gene Sanger sequencing) were regarded as negative controls. Complex indel detection by INDELseek was performed on BWA-MEM (version 0.7.7) alignments of MiSeq NGS data of full *BRCA1* and *BRCA2* genes [6]. The definition of full *BRCA1* and *BRCA2* genes, sequencing methods, analysis methods and partial results were reported previously [6].

### Somatic complex indel detection in myeloid neoplasms

Twenty-two archival DNA samples were retrieved in Hong Kong Sanatorium & Hospital from May 12, 2014 to February 3, 2016. Five of the DNA samples carried somatic pathogenic *CALR* or *JAK2* complex indels and were regarded as positive controls. Remaining seventeen DNA samples of healthy adults with normal complete blood profile were regarded as negative controls as described [16]. Ten core-binding factor leukemia DNA samples were retrieved from Queen Mary Hospital, Hong Kong from January 2003 to December 2014 as a discovery cohort of *KIT* exon 8 mutations. A total of 32 DNA samples were screened by MiSeq sequencing of a 54-gene myeloid NGS gene panel as described [16, 17]. Complex indel detection by INDELseek was performed on BWA-MEM (version 0.7.7) alignments of MiSeq NGS data of *CALR, JAK2* and *KIT* (exon 8 only).

### *In silico* engineering of known complex indels to real NGS data

Known complex indels were collected based on VCF files from COSMIC v71 release [22] and dbSNP b146 release [23]. MutationEngineer was developed to engineer mutation into described real NGS data. Input is the variant of interest and NGS read alignments (VCF and SAM formats, respectively) and output is the engineered read alignments (SAM format) for conversion to FASTQ sequencing reads. Variant allele frequency of complex indel was engineered to be 100%. Each complex indel was engineered as a separate set of FASTQ reads, which were analyzed in the same way as real NGS data. Variants were annotated using Variant Effect Predictor version 75 [24]. Semi-simulation was performed on a Cray XC30 supercomputer.

### Orthogonal validation

*BRCA1* and *BRCA2* complex indels were confirmed by conventional PCR and Sanger sequencing [6]. *CALR* and *JAK2* complex indels were confirmed by conventional PCR and Sanger sequencing or conventional PCR fragment analysis [5, 16]. *KIT* exon 8 complex indels were confirmed by conventional PCR fragment analysis [16] or microfluidic PCR followed by MiSeq sequencing [25]. The primers used in these validation studies were different from those used in the original NGS datasets (Additional file 1: Table S3).

### Reference sequences

Human reference genome sequence: GRCh37/hg19, *BRCA1*: NM_007294.3, *BRCA2*: NM_000059.3, *TP53*: NM_000546.4, *PTEN*: NM_000314.4, *CALR*: NM_004343.3, *KIT*: NM_000222.2 and *JAK2*: NM_004972.3. Variants were described according to the recommendations of Human Genome Variation Society (HGVS) [26]. Variant descriptions were checked by Mutalyzer Name Checker [26].

## Availability and requirements

The authors declare that the data supporting the findings of this study are available within the article and its supplementary information files. Primary sequencing data of clinical samples are available on request from the corresponding author ESKM. The sequencing data are not publicly available due to them containing information that could compromise research participant privacy or consent.

Project name: INDELseek

Project home page: https://github.com/tommyau/indelseek/

Operating system(s): Unix-like (Linux, Mac OS X)

Programming language: Perl

Other requirements: SAMtools 1.3 or higher

License: free for non-profit and academic use.

## Additional files

Additional file 1: Table S1. Putative complex indels curated from GIAB high-confidence variant calls (*n* = 160). **Table S2.** Closely spaced SNV *in trans* curated from GIAB high-confidence variant calls (*n* = 26). **Table S3.** Primer sequences for orthogonal validation. (PDF 564 kb)
Additional file 2:
**Figures S1-S14.** Complex indels detected in samples 1–14 and orthogonal validation. (PDF 1637 kb)
