# Supplementary material for: INDELseek: detection of complex insertions and deletions from next-generation sequencing data
Source: BMC Genomics. 2017 Jan 5;18:16. doi: 10.1186/s12864-016-3449-9 (PMC5217656; doi:10.1186/s12864-016-3449-9)
Supplement: Additional file 1: Table S1. — Putative complex indels curated from GIAB high-confidence variant calls (n = 160). Table S2. Closely spaced SNV in trans curated from GIAB high-confidence variant calls (n = 26). Table S3. Primer sequences for orthogonal validation. (PDF 564 kb) [file 12864_2016_3449_MOESM1_ESM.pdf]

Table S1. Putative complex indels curated from GIAB high-confidence variant calls (n=160).

Number	Genomic region
1	1:889158-889159
2	1:24201919-24201920
3	1:28209362-28209366
4	1:40533315-40533326
5	1:62740446-62740449
6	1:76344705-76344711
7	1:100598866-100598867
8	1:117487710-117487711
9	1:201181217-201181223
10	1:204413297-204413299
11	1:248059703-248059712
12	1:248112762-248112771
13	10:8007559-8007560
14	10:11805354-11805357
15	10:26357747-26357748
16	10:65225244-65225245
17	10:73115941-73115942
18	10:91371445-91371446
19	10:105218252-105218254
20	10:118231360-118231363
21	10:124271589-124271595
22	11:244106-244115
23	11:244167-244171
24	11:280816-280817
25	11:406473-406483
26	11:2435946-2435956
27	11:7847466-7847472
28	11:58207203-58207204
29	11:60620585-60620590
30	11:77790653-77790655
31	11:123777497-123777498
32	11:128782002-128782012
33	12:4881766-4881775
34	12:48919659-48919660
35	12:50749221-50749227
36	12:51740387-51740416
37	12:76461137-76461145
38	12:117465857-117465867
39	12:132682481-132682484
40	13:41767338-41767341
41	13:46108853-46108854
42	13:96540141-96540143
43	13:111154058-111154061
44	13:111155773-111155779
45	14:23944505-23944514
46	15:29418573-29418574

47	15:41483631-41483636
48	15:43817404-43817406
49	15:65370350-65370353
50	15:79231518-79231523
51	15:93198671-93198684
52	15:98504322-98504326
53	15:100246936-100246942
54	16:56601720-56601722
55	16:57071113-57071114
56	16:81190598-81190601
57	16:82203758-82203768
58	16:89167138-89167140
59	16:89167395-89167404
60	17:8108331-8108339
61	17:41960633-41960634
62	17:42852604-42852610
63	17:43318778-43318779
64	17:53851071-53851078
65	17:72469958-72469966
66	18:30804756-30804758
67	18:54354074-54354076
68	18:56203891-56203898
69	18:56204387-56204410
70	19:3750615-3750622
71	19:4513143-4513144
72	19:8191238-8191248
73	19:8399628-8399635
74	19:11221454-11221457
75	19:14910438-14910440
76	19:17435884-17435887
77	19:20808143-20808149
78	19:40901604-40901614
79	19:44352665-44352666
80	19:44612005-44612014
81	19:49207255-49207257
82	19:51522286-51522291
83	2:3653842-3653844
84	2:21232803-21232804
85	2:28824793-28824803
86	2:29274720-29274730
87	2:71004492-71004494
88	2:215632255-215632256
89	2:216272900-216272907
90	2:220414016-220414019
91	2:220500423-220500426
92	2:228194480-228194481
93	2:232087474-232087475
94	20:1896051-1896060
95	20:2413125-2413126
96	20:60908964-60908969

97	20:62200575-62200576
98	21:30257560-30257568
99	21:45978499-45978503
100	21:46067180-46067184
101	22:39497452-39497454
102	3:15737689-15737698
103	3:58416535-58416538
104	3:66550756-66550762
105	3:124802881-124802888
106	3:190106071-190106074
107	3:194373832-194373833
108	4:1087327-1087338
109	4:5743509-5743512
110	4:6292909-6292915
111	4:79420864-79420870
112	4:169369853-169369862
113	5:41049389-41049397
114	5:41061715-41061716
115	5:75923285-75923294
116	5:120021817-120021819
117	5:139931628-139931629
118	5:139931776-139931779
119	5:150518358-150518359
120	5:176936646-176936650
121	6:4057428-4057429
122	6:13814338-13814340
123	6:27792221-27792228
124	6:31079643-31079644
125	6:31113275-31113276
126	6:39033593-39033602
127	6:43014298-43014299
128	6:80228535-80228541
129	6:89913209-89913218
130	6:101312097-101312104
131	6:112508769-112508770
132	6:132271952-132271959
133	6:150210681-150210685
134	6:154414563-154414573
135	6:158735087-158735090
136	6:166755975-166755979
137	7:1533556-1533566
138	7:1586653-1586662
139	7:4802029-4802035
140	7:21582963-21582964
141	7:21628237-21628242
142	7:37934146-37934147
143	7:47913579-47913580
144	7:55991292-55991293
145	7:75441108-75441111
146	7:150556055-150556056

147	8:13356818-13356819
148	8:124664873-124664874
149	8:125107201-125107207
150	8:144399885-144399886
151	8:144649541-144649550
152	8:144654249-144654255
153	8:144654582-144654594
154	9:72897434-72897440
155	9:97369149-97369151
156	9:116770776-116770785
157	9:132636031-132636033
158	9:138586966-138586967
159	X:69749852-69749853
160	X:82764040-82764042

Table S2. Closely spaced SNV *in trans* curated from GIAB high-confidence variant calls (n=26).

Number	Genomic region
1	1:155033308-155033317
2	10:6066195-6066200
3	10:72517830-72517837
4	10:134736044-134736052
5	11:5906203-5906205
6	11:10555586-10555589
7	11:125617606-125617610
8	12:91449984-91449990
9	14:24545366-24545375
10	14:93118668-93118669
11	15:66629394-66629403
12	16:81180988-81180995
13	17:2203167-2203175
14	17:11835321-11835331
15	19:1009550-1009585
16	19:18497137-18497141
17	19:35770056-35770064
18	2:160808075-160808076
19	2:231036860-231036866
20	21:19666901-19666910
21	21:41137503-41137507
22	3:58191266-58191274
23	6:26365586-26365595
24	6:32805306-32805307
25	7:38431430-38431436
26	9:117846570-117846580

Table S3. Primer sequences for orthogonal validation.

Application	Sequences
Sanger sequencing for *BRCA1* c.4046_4049delinsAGG	5'-GTAATATTGGCAAAGGCATCT-3',<br>5'-TAAAATGTGCTCCCCAAAAGCA-3'
Sanger sequencing for *BRCA2* c.4467_4474delinsTGTTTTT	5'-AACGGACTTGCTATTTACTGA-3',<br>5'-AGTACCTTGCTCTTTTTCATC-3'
Sanger sequencing for *BRCA2* c.8400_8402delinsAAAA	5'-ATATTTTTAAGGCAGTTCTAGA-3',<br>5'-TTACACACACCAAAAAAGTCA-3'
Sanger sequencing for *CALR* exon 9 mutations	5'-CAGGTCAAGTCTGGCACCAT-3',<br>5'-ACAGAGACATTATTTGGCGCG-3'
Sanger sequencing for *JAK2* exon 12 mutations	5'-CTCCTCTTTGGAGCAATTCA-3',<br>5'-GAGAACTTGGGAGTTGCGATA-3'
Conventional PCR fragment analysis of for *CALR* exon 9 mutations	5'-NED-CAGGTCAAGTCTGGCACCAT-3',<br>5'-ACAGAGACATTATTTGGCGCG-3'
Conventional PCR fragment analysis for *KIT* exon 8 mutations	5'-ACTCTGACATATGGCCATTTCTGTTTTC-3',<br>5'-TGTAAAACGACGGCCAGTAAACATATTT-GAAATTCAAGTGAATTGCA-3',<br>5'-6-FAM-TGTAAAACGACGGCCAGT-3'
Microfluidic PCR and MiSeq sequencing for *KIT* exon 8 mutations	5'-ACACTCTTTCCCTACACGACGCTCTTCC-GATCTAAACATATTTGAAATTCAAGTGAATTGCA-3',<br>5'-GTGACTGGAGTTCAGACGTGTGCTCTTC-CGATCTACTCTGACATATGGCCATTTCTGTTTTC-3'
