# Supplementary material for: INDELseek: detection of complex insertions and deletions from next-generation sequencing data
Source: BMC Genomics. 2017 Jan 5;18:16. doi: 10.1186/s12864-016-3449-9 (PMC5217656; doi:10.1186/s12864-016-3449-9)

Figure S1. Complex indel detected in sample 1 and orthogonal validation. (**A**) IGV screenshot of representative original NGS reads. (**B**) Validation by Sanger sequencing.

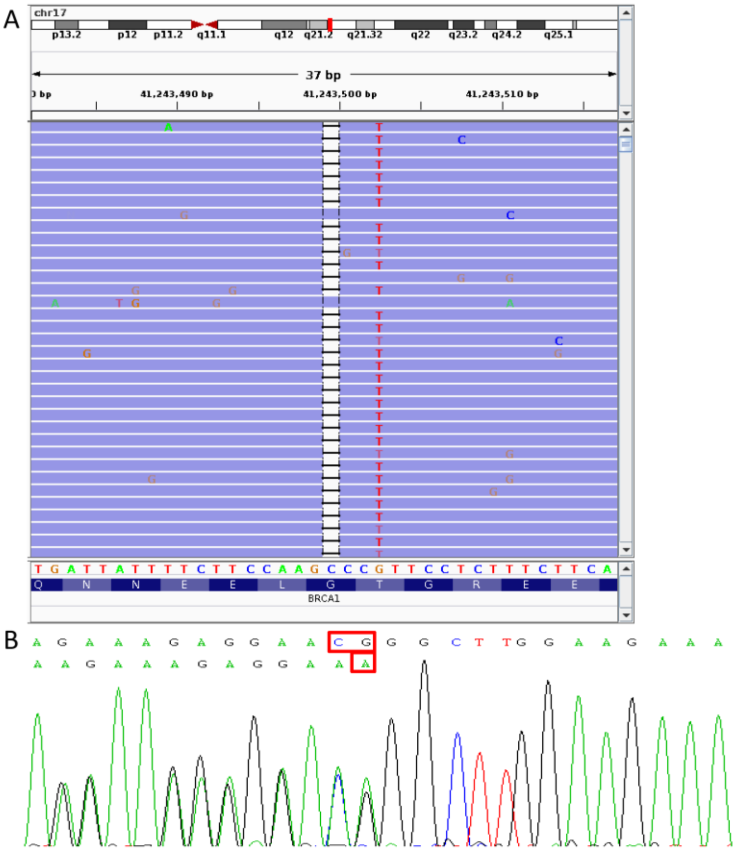

Figure S2. Complex indel detected in sample 2 and orthogonal validation. (A) IGV screenshot of representative original NGS reads. (B) Validation by Sanger sequencing.

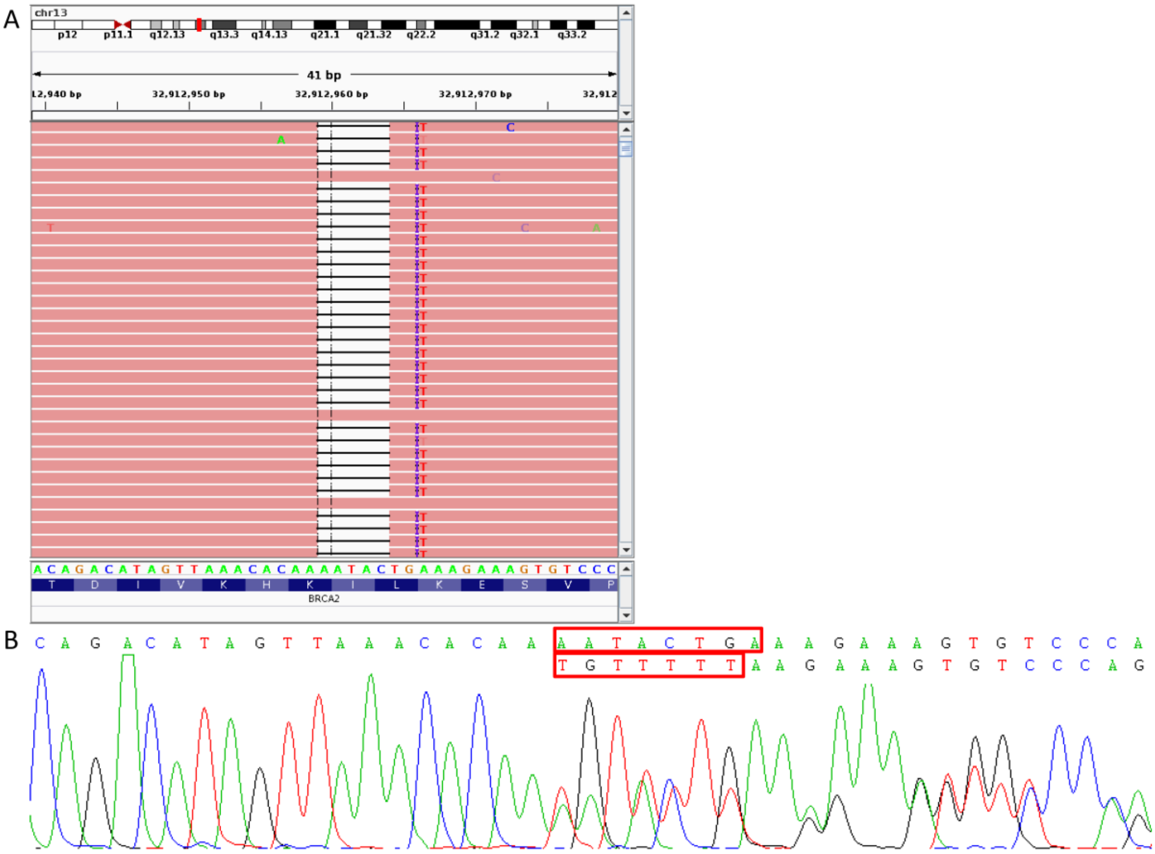

Figure S3. Complex indel detected in sample 3 and orthogonal validation. (**A**) IGV screenshot of representative original NGS reads. (**B**) Validation by Sanger sequencing.

A

B

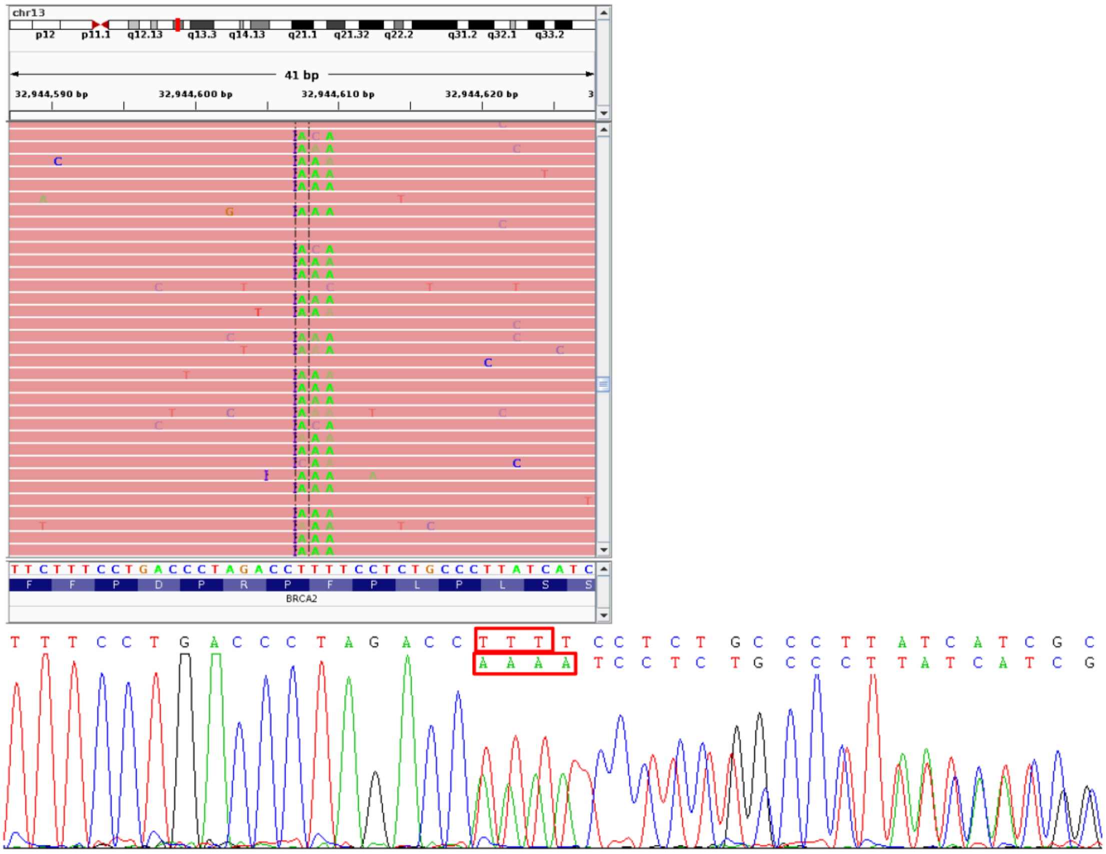

Figure S4. Complex indel detected in sample 4 and orthogonal validation. (**A**) IGV screenshot of representative original NGS reads. (**B**) Validation by Sanger sequencing.

A

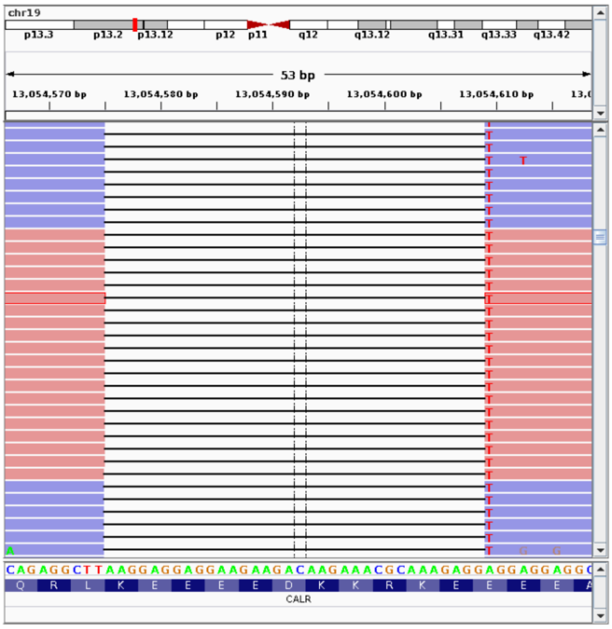

B

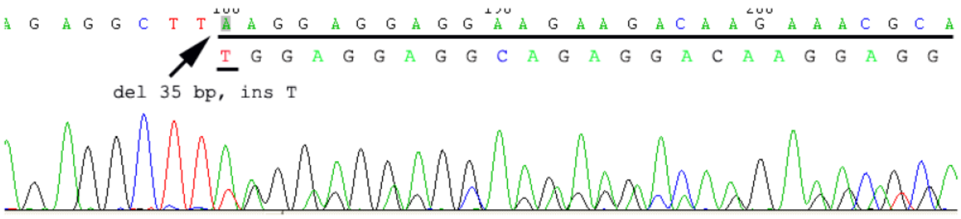

Figure S5. Complex indel detected in sample 5 and orthogonal validation. (**A**) IGV screenshot of representative original NGS reads. (**B**) Validation by Sanger sequencing.

A

B

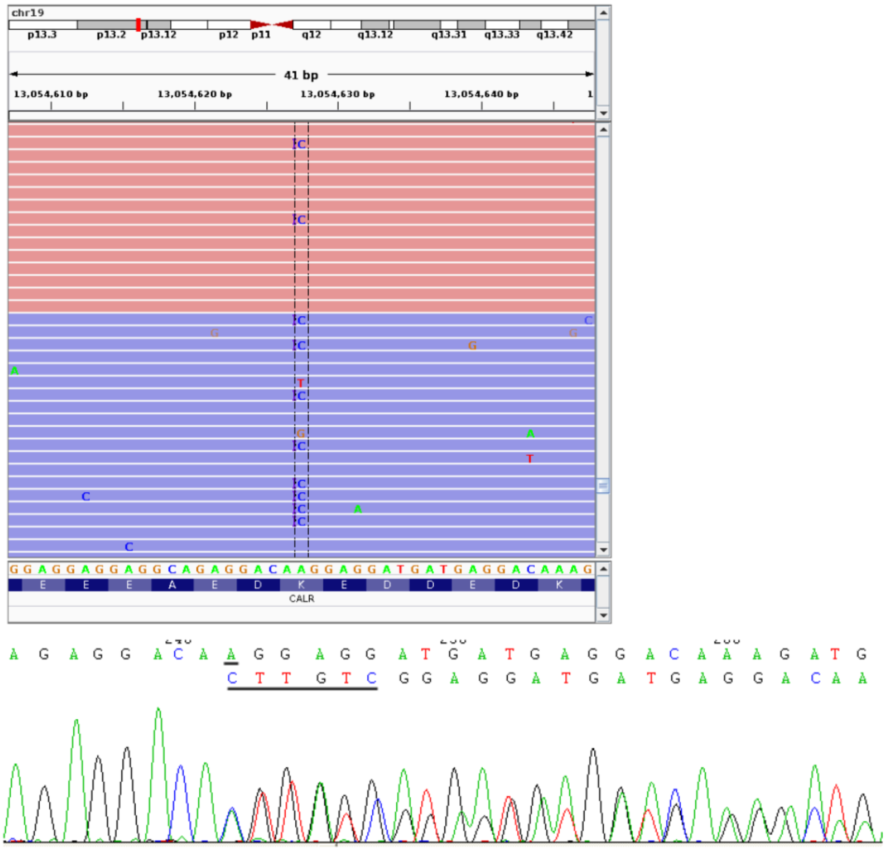

Figure S6. Complex indel detected in sample 6 and orthogonal validation.

(A) IGV screenshot of representative original NGS reads. (**B**) Validation by Sanger sequencing.

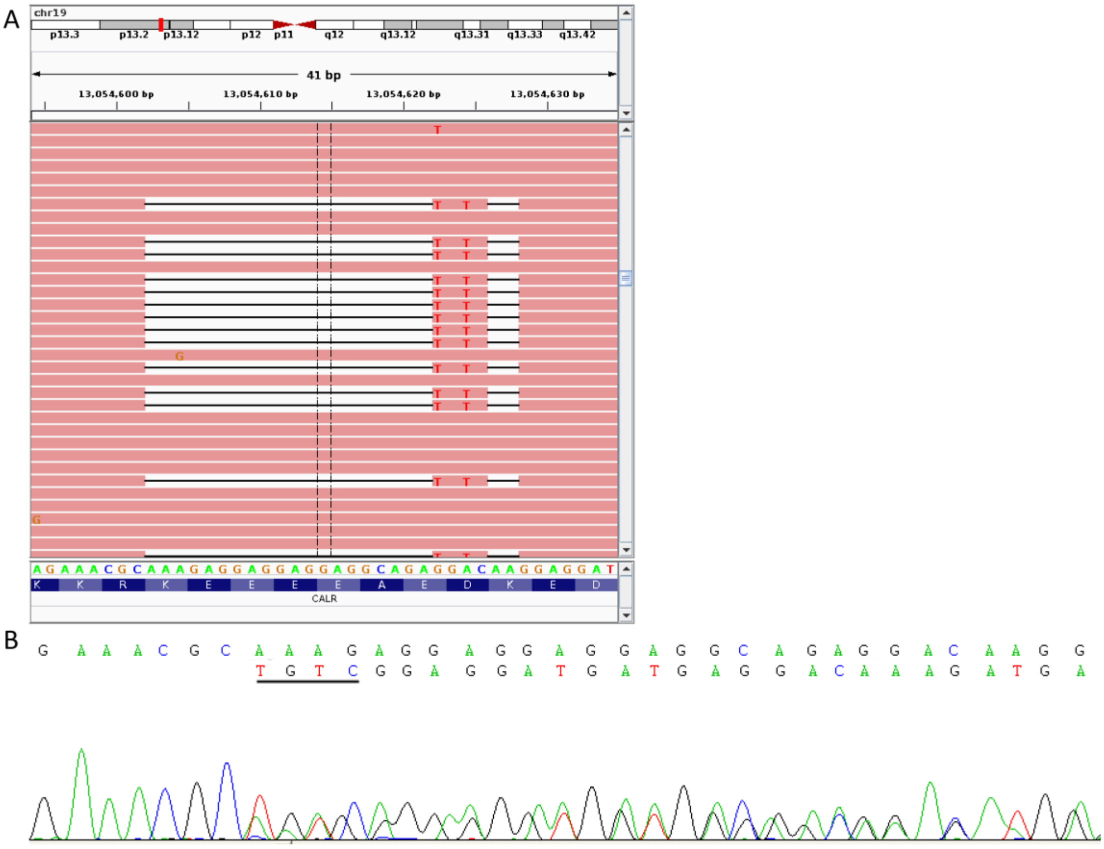

Figure S7. Complex indel detected in sample 7 and orthogonal validation. (**A**) IGV screenshot of representative original NGS reads. (**B**) Validation by conventional PCR fragment analysis. Wild-type (WT) and mutant (asterisk) fragments were shown.

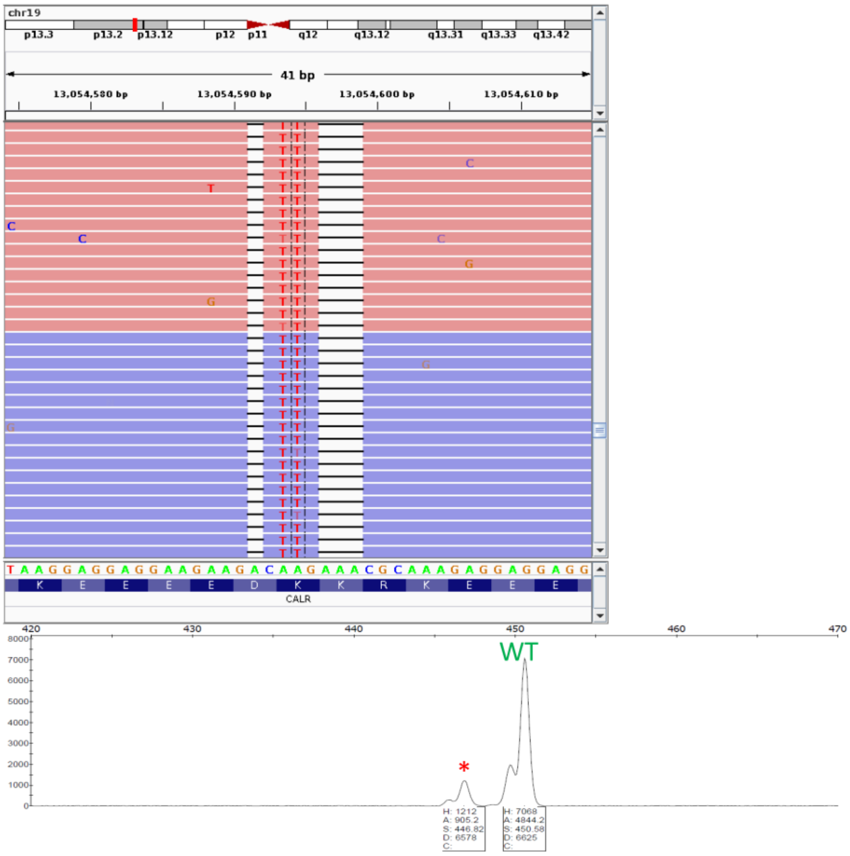

Figure S8. Complex indel detected in sample 8 and orthogonal validation. (**A**) IGV screenshot of representative original NGS reads. (**B**) Validation by Sanger sequencing.

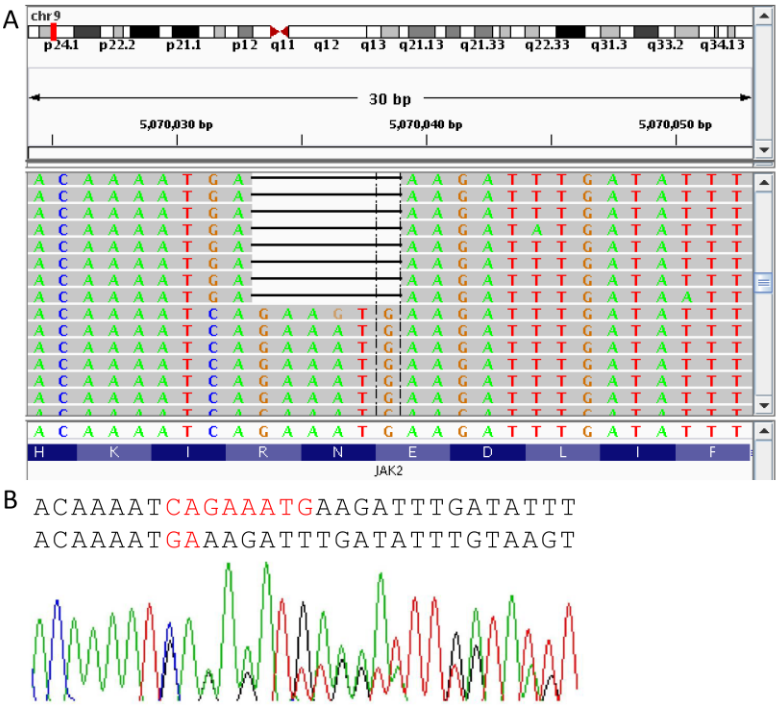

Figure S9. Complex indel detected in sample 9 and orthogonal validation. (**A**) IGV screenshot of representative original NGS reads. (**B**) IGV screenshot of representative validation NGS reads.

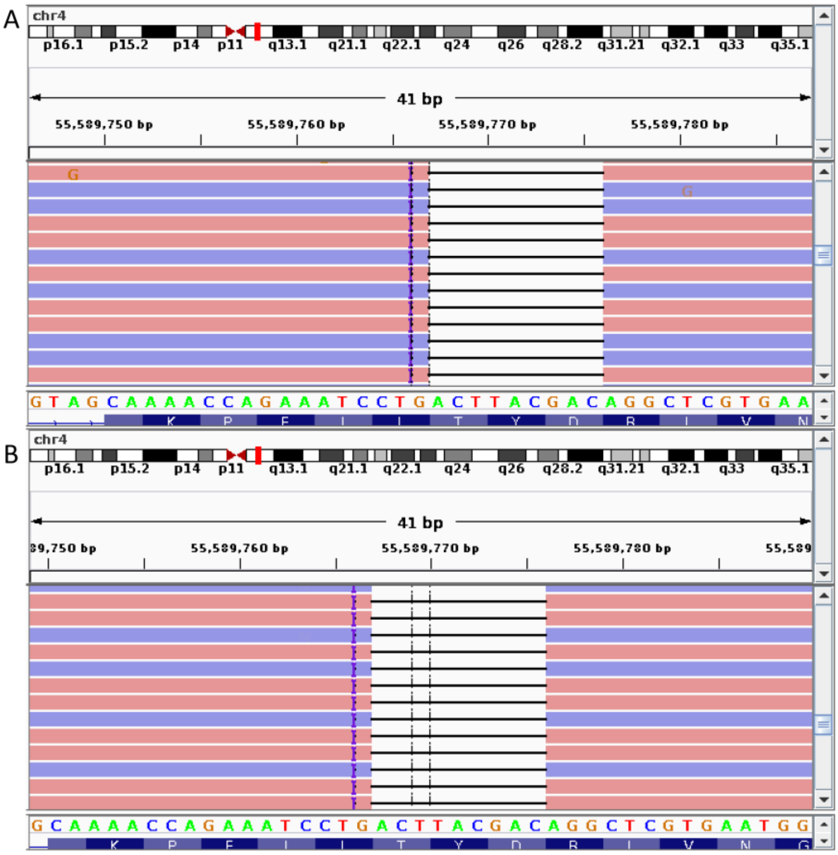

Figure S10. Complex indels detected in sample 10 and orthogonal validation. (**A-D**) IGV screenshot of representative original NGS reads of four complex indels c.1248_1256delinsTTTCCG, c.1249_1258delinsGGATGGAACT, c.1250_1258delinsAACCTC and c.1251_1258delinsCTCCT, respectively. (**E-H**) IGV screenshot of representative validation NGS reads for the corresponding compelx indels shown in (**A-D**).

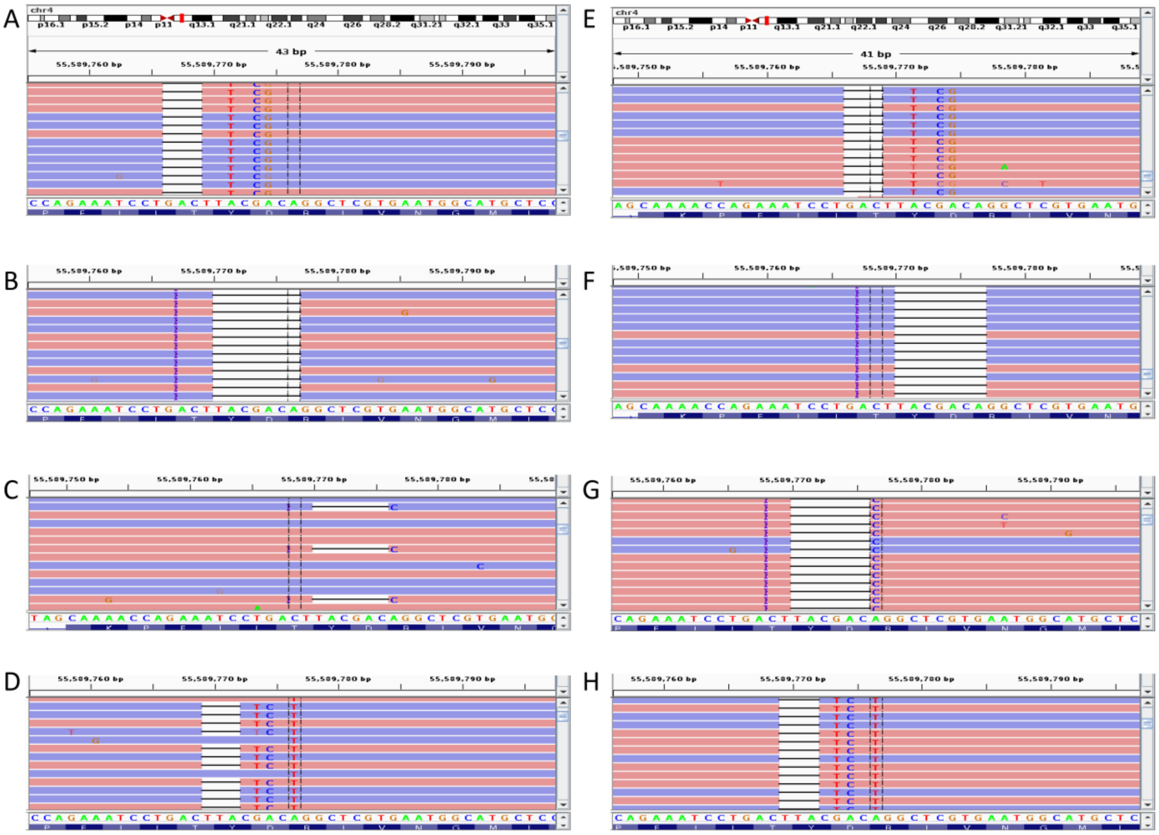

Figure S11. Complex indels detected in sample 11 and orthogonal validation.

(A-B) IGV screenshot of representative original NGS reads of two complex indels c.1250_1256delinsT and c.1251_1257delinsAACA, respectively. (**C**) Wild-type (WT) and mutant (single asterisk for c.1250_1256delinsT, double asterisk for c.1251_1257delinsAACA) fragments were shown.

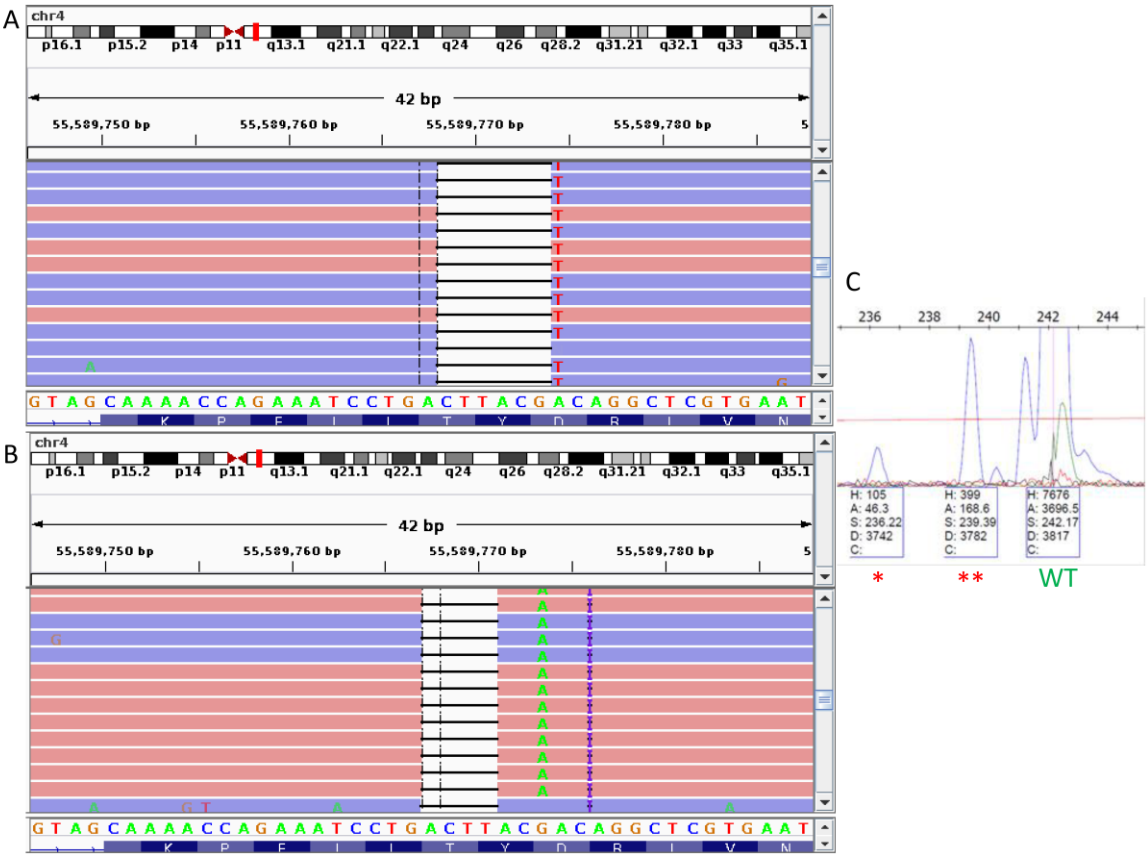

Figure S12. Complex indel detected in sample 12 and orthogonal validation. (**A**) IGV screenshot of representative original NGS reads. (**B**) IGV screenshot of representative validation NGS reads.

A

B

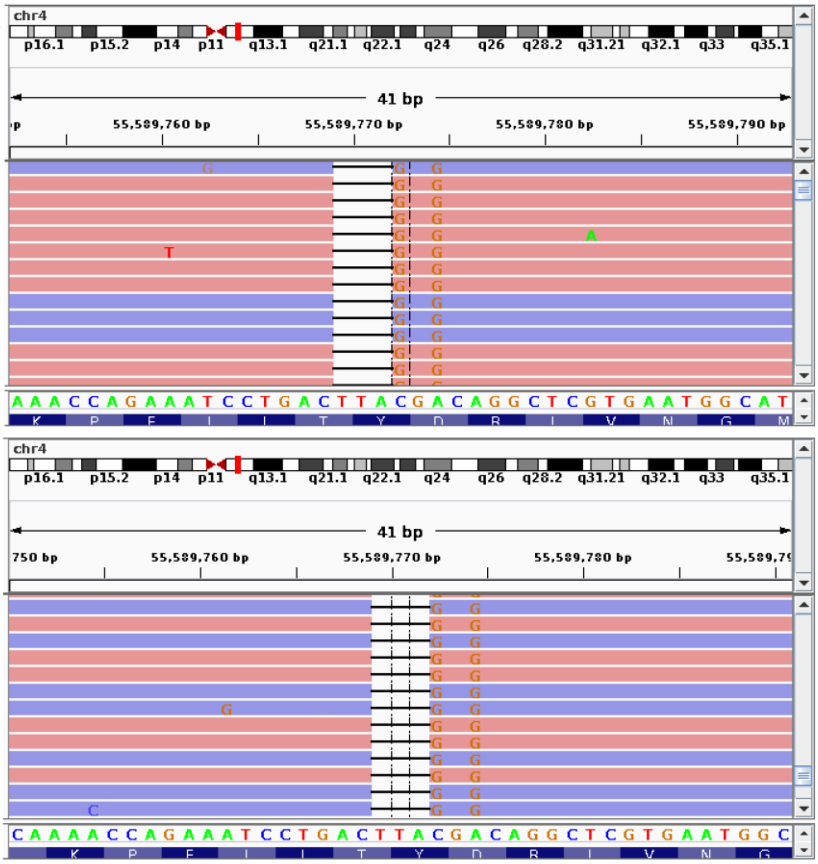

Figure S13. Complex indel detected in sample 13 and orthogonal validation. (**A**) IGV screenshot of representative original NGS reads. (**B**) IGV screenshot of representative validation NGS reads.

A

B

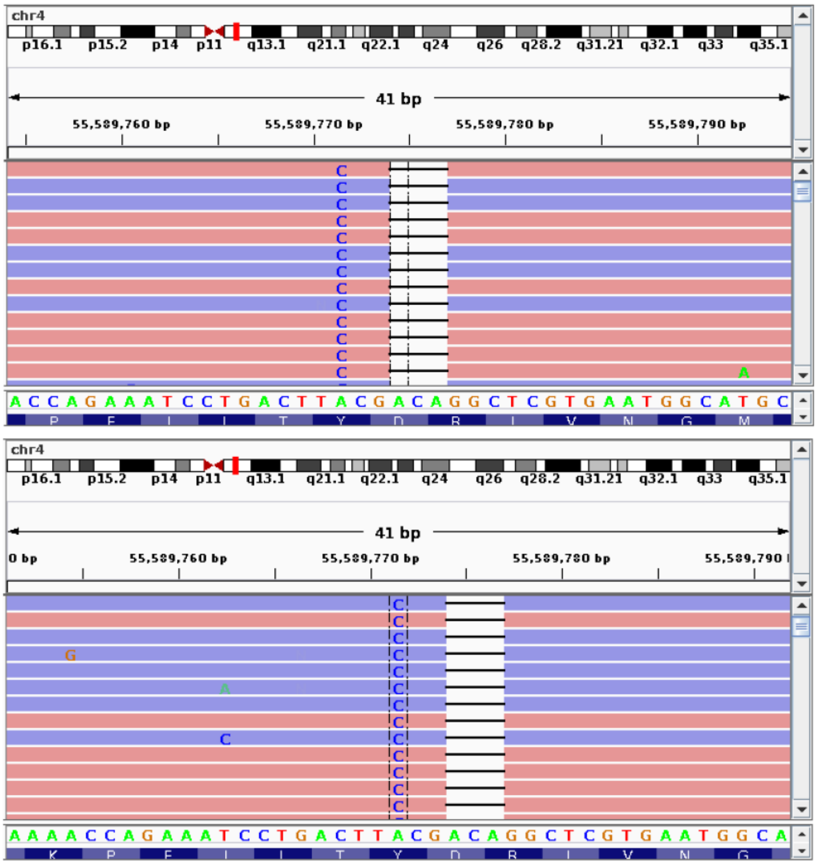

Figure S14. Complex indel detected in sample 14 and orthogonal validation. (**A**) IGV screenshot of representative original NGS reads. (**B**) IGV screenshot of representative validation NGS reads.

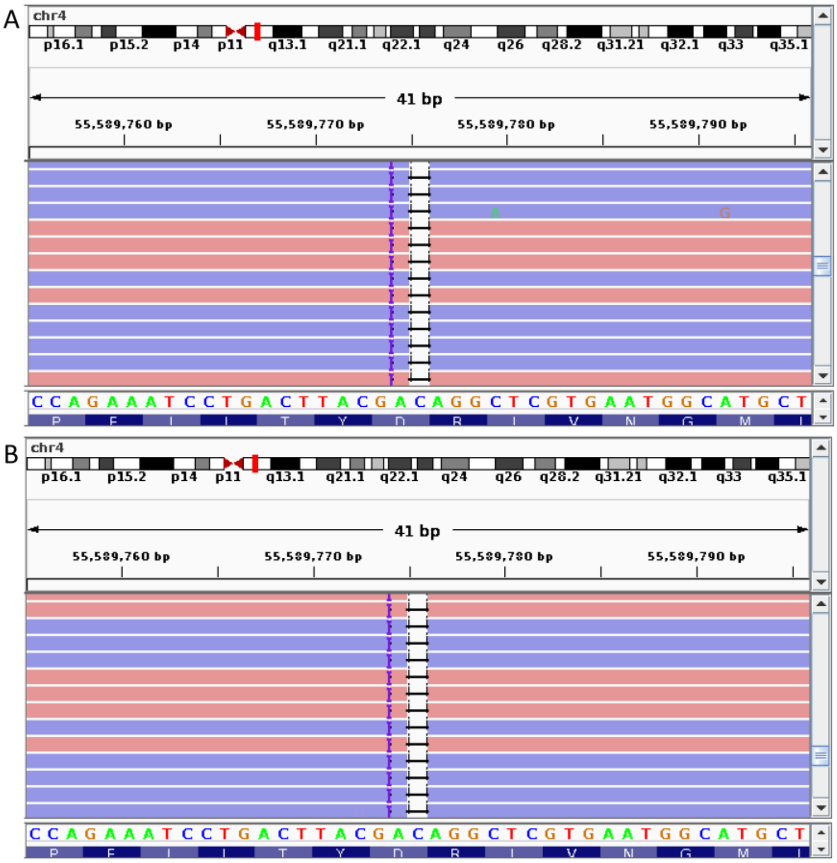

Supplement: Additional file 2: — Figures S1-S14. Complex indels detected in samples 1–14 and orthogonal validation. (PDF 1637 kb) [file 12864_2016_3449_MOESM2_ESM.pdf]
